# Multimodal Mass Spectrometry Imaging of an Osteosarcoma Multicellular Tumour Spheroid Model to Investigate Drug-Induced Response

**DOI:** 10.3390/metabo14060315

**Published:** 2024-05-29

**Authors:** Sophie M. Pearce, Neil A. Cross, David P. Smith, Malcolm R. Clench, Lucy E. Flint, Gregory Hamm, Richard Goodwin, James I. Langridge, Emmanuelle Claude, Laura M. Cole

**Affiliations:** 1 Centre for Mass Spectrometry Imaging, Biomolecular Sciences Research Centre, Sheffield Hallam University, Howard Street, Sheffield S1 1WB, UK; s.pearce1@shu.ac.uk (S.M.P.); n.cross@shu.ac.uk (N.A.C.); d.p.smith@shu.ac.uk (D.P.S.); m.clench@imagtech.co.uk (M.R.C.); 2 Imaging and Data Analytics, Clinical Pharmacology and Safety Sciences, BioPharmaceuticals R&D, AstraZeneca, The Discovery Centre (DISC), Biomedical Campus, 1 Francis Crick Ave, Trumpington, Cambridge CB2 0AA, UK; lucy.flint@astrazeneca.com (L.E.F.); gregory.hamm@astrazeneca.com (G.H.); richard.goodwin@astrazeneca.com (R.G.); 3 Waters Corporation, Stamford Avenue, Altrincham Road, Wilmslow, Cheshire SK9 4AX, UK; james_langridge@waters.com (J.I.L.); emmanuelle_claude@waters.com (E.C.)

**Keywords:** chemotherapy, mass spectrometry imaging, DESI-MSI, MALDI-IHC, imaging mass cytometry, doxorubicin, multicellular tumour spheroid, osteosarcoma, 3D cell culture, chemoresistance

## Abstract

A multimodal mass spectrometry imaging (MSI) approach was used to investigate the chemotherapy drug-induced response of a Multicellular Tumour Spheroid (MCTS) 3D cell culture model of osteosarcoma (OS). The work addresses the critical demand for enhanced translatable early drug discovery approaches by demonstrating a robust spatially resolved molecular distribution analysis in tumour models following chemotherapeutic intervention. Advanced high-resolution techniques were employed, including desorption electrospray ionisation (DESI) mass spectrometry imaging (MSI), to assess the interplay between metabolic and cellular pathways in response to chemotherapeutic intervention. Endogenous metabolite distributions of the human OS tumour models were complemented with subcellularly resolved protein localisation by the detection of metal-tagged antibodies using Imaging Mass Cytometry (IMC). The first application of matrix-assisted laser desorption ionization–immunohistochemistry (MALDI-IHC) of 3D cell culture models is reported here. Protein localisation and expression following an acute dosage of the chemotherapy drug doxorubicin demonstrated novel indications for mechanisms of region-specific tumour survival and cell-cycle-specific drug-induced responses. Previously unknown doxorubicin-induced metabolite upregulation was revealed by DESI-MSI of MCTSs, which may be used to inform mechanisms of chemotherapeutic resistance. The demonstration of specific tumour survival mechanisms that are characteristic of those reported for in vivo tumours has underscored the increasing value of this approach as a tool to investigate drug resistance.

## 1. Introduction

The pharmaceutical industry routinely conducts animal studies as an integral component of drug discovery and development, employing them to assess key properties, such as absorption, distribution, metabolism, excretion, and pharmacokinetic profiles, and to perform proof of concept studies. This standard practice often results in the identification of candidate drugs deemed unsuitable, leading to the termination of their progression [1]. Given the resource-intensive nature of animal studies and the consequential attrition rate [2], there exists a critical need for innovative approaches that can enhance the efficiency of drug discovery. This demand becomes increasingly crucial when considering diseases characterized by poor prognoses, high rates of secondary malignancies, and refractory disease. Osteosarcoma (OS), the most prevalent type of bone cancer in children [3], represents a typical example of this clinical challenge. To address this challenge, there is a growing interest in the application of 3D cell culture tumour models that offer superior clinical accuracy compared to conventional in vitro early drug discovery studies. An in vitro tumour model with enhanced clinical translatability has the capacity to pre-emptively identify candidate drugs that would otherwise progress to animal studies only to be later deemed unsuitable. By implementing such models early in the drug discovery process, there exists the potential for the identification of viable drug candidates to be advanced, leading to a more efficient and cost-effective drug development pipeline. The work addresses a recently identified key challenge for the advancement of the 3Rs (replace, reduce, and refine) agenda in animal experimentation, namely, the failure of these innovative approaches to become widely adopted in the field. This has been attributed to a lack of evidence, compatibility, and the inherent difficulty of integration into long-established practices [4]. The barriers hindering their widespread adoption in established fields is therefore slowing down the integration of 3R-adherent techniques into conventional practices.

This study employs Multicellular Tumour Spheroids (MCTSs) as the primary 3D cell culture model [5] to investigate drug-induced responses. The formation of MCTSs involves seeding and subsequent aggregation of single-cell suspensions into low-adhesion plates, facilitating cell-to-cell adhesion and the development of spheroids [6]. Additionally, investigations were conducted using a larger aggregoid osteosarcoma model formed by the culture of isogenic cells in 3D matrices, specifically alginate spheres [7,8,9]. In the aggregoid model, single cells proliferate within the spheres, forming spheroids by proliferation as opposed to aggregation. Following sufficient proliferation, dissolving the alginate matrix leads to the subsequent release and aggregation of spheroids, resulting in the formation of the aggregoid model—a scaffold-free 3D model. The study reported here focuses on the investigations of MCTSs subjected to doxorubicin (Dox) treatments.

To investigate the drug-induced response of the 3D cell culture tumour models, advanced mass spectrometry imaging techniques were employed, including the DESI-XS multi-reflecting time-of-flight (MRT) mass spectrometry imaging system to assess the interplay between metabolic and cellular pathways in response to chemotherapeutic intervention. The high mass resolution capabilities of the recently developed MRT mass spectrometry technology arise from the use of gridless ion mirrors, which facilitate high-energy focusing, enabling multiple reflections of ions and an effective 47 m time-of-flight path length [10]. This increased capability enhances ion separation and provides an increased comprehensive analysis of the molecular landscape. Further to investigate drug-induced responses within the 3D cell culture tumour models, endogenous metabolite desorption electrospray ionisation–mass spectrometry imaging (DESI-MSI) of the human osteosarcoma tumour models was performed using an Orbitrap mass spectrometer to analyse the impact of increasing acute doses of a chemotherapeutic agent, doxorubicin, on spatially resolved molecular distributions within the tumour models. The atmospheric pressure (AP) ionisation technique, DESI [11], allowed for an in situ high-capacity label-free molecular distribution analysis [12] with limited sample preparation. The AP ionisation capabilities of DESI are executed by the ionisation of tissue surface molecules upon impact of the ESI-charged droplets onto the microtissue surface before ions are introduced into the vacuum MS system [11]. Encompassing a multi-modal imaging approach, drug-induced response was also investigated by matrix-assisted laser desorption ionization–immunohistochemistry mass spectrometry imaging (MALDI-IHC MSI). This is a recent development in the field of mass spectrometry imaging [13] that has unlocked highly multiplexing capabilities with absolute confirmatory protein identifications and without the usual roadblocks faced in multiplexing spatially resolved protein analysis. The technique is based on antibody probing with photocleavable mass tags (PC-MTs) (AmberGen Inc., Watertown, MA, USA) to stain tissues. A specific antibody binds to the protein of interest, which is bound to a photocleavable linker and bound to a peptide mass reporter [13]. Following staining, the technique capitalises on the photocleavable site of the compound design, whereby upon exposure to UV light, the peptide mass reporter is cleaved and available for targeted MSI detection. Matrices are applied and MALDI-MSI can be carried out in the conventional way, whereby a pulsed laser beam is rastered across the slide, ablating the matrix molecules from the surface of the sample, and the analyte molecules, i.e., the photocleaved peptide ions of the mass reporters, are desorbed into the mass spectrometer. The distinctive capability of this approach is the fact that the mass reporters are synthesised to be equipped with a specific peptide and amino acid sequence, therefore offering the capability for the simultaneous application of hundreds of antibody probes, each presented with unique mass reporters, which can be easily resolved and detected by mass spectrometry. MALDI-IHC MSI therefore allows for a previously unheard of highly multiplexed spatially resolved protein localisation imaging [14], given that it is not challenged with the typical limitations of targeted highly multiplexed imaging, e.g., fluorophore excitation and emission spectra overlap, which only allow for around three to five antibodies to stain a single given tissue section [15].

In parallel, to complement MALDI-IHC analyses, a subcellular protein localisation analysis was conducted using Imaging Mass Cytometry (IMC) by time-of-flight; in this case, metal-tagged antibodies were imaged at a subcellular 1 μm spatial resolution using the Hyperion Imaging System. In this study, IMC analyses were able to show single-cell-level protein localisation to compliment endogenous metabolite and lipid molecular mapping. This approach further supports the multi-modal depth of analysis of the OS tumour models and facilitates the investigation of drug-induced responses and chemotherapeutic resistance.

The clinical relevance of a poor osteosarcoma prognosis lies within the current treatment options for osteosarcoma: neoadjuvant chemotherapy and surgical resection, followed by adjuvant chemotherapy [16]. Since they were first introduced following the Multi-Institutional Osteosarcoma Study (MIOS) clinical trial [17], chemotherapeutics predominantly include the MAP drug combination (methotrexate, doxorubicin, and cisplatin), which remains the current standard of care for chemotherapy options, as well as the cytotoxic agent ifosfamide [18]. Doxorubicin treatment is investigated within this study, as it is a chemotherapeutic of the anthracycline class, which has had wide clinical oncology application since it was initially isolated from *Streptomyces peucetius* var. *caesius* [19]. Doxorubicin, being a topoisomerase II inhibitor, induces DNA strand breakage and thus inhibits DNA replication and inhibition of topoisomerases [20]. Doxorubicin is also known to disrupt mitochondrial function, increase free radical generation, and induce oxidative damage [21]. However, the mechanisms of action of doxorubicin drug resistance influenced by metabolome changes in osteosarcoma remain largely unknown. Similarly, resistant and non-responding osteosarcoma is largely poorly understood. This study therefore has a wide scope for uncovering mechanisms of action and resistance, which may inform improved treatment regimens and novel therapeutics.

It is important to consider and distinguish between the two key types of drug resistance: intrinsic and acquired resistance. Intrinsic resistance is demonstrated by treatment-naïve cancer cells with the ability to proliferate upon the initial introduction of chemotherapeutic agent, whilst sensitive cells are at the same time undergoing cell death caused by chemotoxicity. This intrinsic resistance is understood to be generally attributed to pre-existing mutations or intrinsic/endogenous activation of signalling pathways that favour survival [22]. Conversely, acquired resistance has been observed to develop following therapeutic intervention, and is associated with a common, gradual reduction in treatment efficacy over time [23]. Acquired resistance can be evidenced by the activation of secondary proto-oncogenes as newly emerged driver genes following chemotherapy, drug-induced mutations, or a change in the expression of proteins. In particular, alterations in protein abundance, where the protein is regulated by the chemotherapeutic agent and consequently ceases to be controlled, leading to a loss in manipulation of the protein as an active drug target. Acquired resistance can also be evidenced by changes in the regulation of metabolic pathways and potential resulting changes in the tumour microenvironment [24].

A known resistance mechanism to doxorubicin treatment thought to play a role in osteosarcoma resistance incorporates a modality of intracellularly accumulated drug efflux. This resistance mechanism acts to decrease intracellular drug accumulation and subsequent cytotoxicity via increased drug elimination. Studies that have supported this idea have shown an upregulation of ABCB1 in doxorubicin induced resistance. ABCB1 is known to act as a membrane efflux pump that eliminates drug substrates [25,26]. These ideas have been further developed by the recent discovery of a link between chemotherapy and immune sensitivity, whereby the ABCA1/ABCB1 ratio determines doxorubicin sensitivity [27]. Decreased ABCB1 levels and increased ABCA1 levels are characteristics of doxorubicin-sensitive osteosarcoma that are interestingly evidenced not only in acquired resistant cells over long-term treatments but also resistant, treatment-naïve 3D constructs compared to their 2D monolayer cultured counterparts. These results once again underscore the importance of the development of more established tools to investigate drug-induced responses and resistance in models that possess increased clinical translatability, i.e., 3D tumour models, which exhibit resistance mechanisms attributed to their accurate representation of an in vivo environment where cell-to-cell adhesion is considered. The discussed observations presented a constitutively activated Ras/Akt/mTOR pathway in the characterised resistant OS models [27]. These findings can be linked to data presented here, which also have the added dimension of spatial resolution to delineate changes in regulation within the tumour models. Additionally, increased accumulation of cholesterol in doxorubicin-resistant OS cells was found to be correlated with low ABCA1 expression following a combination of reduced cholesterol efflux and increased cholesterol synthesis [27].

Drug resistance mechanisms have been extensively studied using metabolomics techniques, but chemoresistance in osteosarcoma has not, and the use of imaging technologies as reported here is entirely novel. Similarly, molecular changes attributed to the metastasis of human osteosarcoma are not well understood. However, a metabolomics study that investigated human osteosarcoma cell lines and their increasingly metastatic counterparts, which were developed by in vivo selection and an experimental metastasis evaluation assay [28], revealed a link between inositol metabolism and osteosarcoma metastasis [29]. The inositol pathway metabolites glucose-6 phosphate, myo-inositol 1-phosphate and myo-inositol were significantly decreased in highly metastatic OS cells in comparison to their clonally related low metastatic parental counterparts [29]. Confirmatory experiments demonstrated that upon the introduction of exogenous inositol, IP6, to metastatic cells, a reduction in metastatic capability was observed. The findings therefore demonstrated that by an increase in exogenous inositol levels, glucose metabolism was inhibited, the PI3K/Akt pathway was inhibited and a significant reduction in the phosphorylation of kinases in both PI3K and MAPK signalling pathways, such as pERK1/2, pAkt and pGSK-3a/b, was observed [29]. The study gave an initial insight to understand the metastatic potential of osteosarcoma, which most commonly presents as pulmonary metastases that continue to be the lead cause of osteosarcoma-associated death [30]. MSI has allowed the study of the spatially resolved molecular distribution across the tumour models, informing the development of an early drug discovery tool that maximises the capabilities of a high-resolution label-free spatial distribution technique to simultaneously map hundreds of molecules.

Previous studies investigating the response of 2D osteosarcoma cells to acute doxorubicin treatments in comparison to a novel 3D aggregoid model reported a lack of cytotoxicity observed in the 3D tumour model [7]. Despite this, a discriminatory metabolomic response was observed by MALDI-MSI, underscoring the increased clinical relevance of 3D models compared to 2D models and the value of MSI to uncover drug-induced responses that cannot be observed with conventional dose–response and cytotoxicity assays. It also highlights the importance of an untargeted investigation of the response to inform mechanistic investigations. The application of sophisticated 3D cell culture models in the bioanalytical space is becoming pivotal for elucidating a wider aspect of cellular responses to therapeutic interventions. In this study, we present and discuss the outcomes of our investigation, highlighting the significance of these advanced techniques in unravelling the complex dynamics of drug responses within the context of osteosarcoma, specifically when subjected to doxorubicin treatments.

## 2. Materials and Methods

### 2.1. Osteosarcoma 3D Cell Culture

The human osteosarcoma cell line, SAOS-2 (ATCC, Manasas, VA, USA), was cultured in α-MEM (Minimum Essential Medium Eagle-α modification) supplemented with 10% foetal bovine serum and 1% penicillin–streptomycin (ThermoFisher Scientific, Waltham, MA, USA). Cells were cultured at 37 °C, 5% CO_2_. Multicellular Tumour Spheroids (MCTSs) [31] were cultured to form 3D models by the aggregation of 1 × 10^4^ cells per well in low-adhesion U-bottom plates with the CellStar Cell-Repellent surface (Greiner Bio-One Ltd., Gloucestershire, UK). MCTSs were cultured for a total duration of 96 h.

### 2.2. Chemotherapeutic Agent Treatments

The doxorubicin hydrochloride (Merck KGaA, Darmstadt, Germany) stock solution was prepared in Milli-Q ultra-pure water. The cytotoxic drug solution was added to MCTSs in fresh culture media and dosed at final concentrations of 1 μM, 5 μM, 20 μM and 100 μM. Doxorubicin doses were administered at 48 h following the seeding of the single-cell suspension to allow for the MCTSs to fully form. Doxorubicin was administered for 48 h.

### 2.3. Microtissue Preparation

Upon reaching treatment end points, MCTSs were pooled by treatment groups, n = 10, and transferred into vessels in which the media were discarded, and spheroids were washed 3× with ice-cold phosphate-buffered saline solution (ThermoFisher Scientific, Waltham, MA, USA). MCTSs pooled from treatment groups were embedded (n = 10) in 7.5% hydroxypropyl methylcellulose (HPMC) and 2.5% polyvinylpyrrolidone (PVP) as per the developed method optimal for multi-modal molecular imaging [32]. Embedded MCTSs were cryosectioned (Leica Biosystems, Nussloch, Germany) at 10 μm, immediately thaw-mounted onto poly-L-lysine-coated microscope slides (Merck KGaA, Darmstadt, Germany) or indium tin oxide slides (VisionTek Systems Ltd., Cheshire, UK) for MALDI analyses, and dried with a stream of compressed air to allow for rapid drying of the micro tissues and to avoid metabolite delocalisation. Slide mailers were vacuum-packed and stored at −80 °C [33]. Sectioned microtissues on slides were thawed at room temperature whilst remaining under vacuum to avoid molecular delocalisation effects caused by condensation prior to analysis.

### 2.4. 3D Cell Viability Assay

SAOS-2 MCTSs were subjected to a CellTiter-Glo 3D Cell Viability Assay (Promega, Madison, WI, USA) following 48 h of doxorubicin exposure. The Glo-Reagent was added at a ratio of 1:1 and spheroids were incubated according to the manufacturer’s protocol [34]. Relative Luminescence Units (RLUs) were read by the ClarioStar Microplate Reader (BMG Labtech, Baden-Württemberg, Germany) as function of the ATP concentration. Media–drug solutions in the absence of cells were employed as negative controls, from which the background was subtracted to calculate normalised ATP read outs. Normalised ATP read outs of untreated MCTSs were subtracted from the treated models to calculate cell viability as a percentage of the controls. A dose–response curve following 48 h of exposure to doses of 1 μM, 5 μM, 20 μM and 100 μM doxorubicin, n = 6, was graphed where the inhibitor vs. normalised response was plotted in a non-linear regression fit using GraphPad Prism version 8.1.1 (GraphPad Prism, Boston, MA, USA).

### 2.5. Desorption Electrospray Ionisation-Mass Spectrometry Imaging (DESI-MSI)

DESI-MSI of the osteosarcoma tumour models was performed using the SELECT SERIES MRT, DESI XS System (Waters Corporation, Wilmslow, UK) in negative ionisation mode. Images were acquired at a 30 μm spatial resolution at a 10 Hz scan speed. The DESI flow rate was operated at 2 μL/min with 95:5 methanol/H_2_O spiked with 100 pg/μL Leucine Enkephalin to provide a continuous internal lock mass. Nitrogen nebulising gas was operated at 10 psi. A mass resolution of 200,000 FWHM was achieved by the MRT operating at a flight path of 47 m [10]. The quadrupole profile was custom set for optimal transmission of low-mass-range metabolites at *m*/*z* 50, 100 and 200. Data were processed using High-Definition Imaging (HDI) Software version 1.7 (Waters Corporation, Wilmslow, UK). Endogenous metabolite DESI-MSI was also performed using a Q-Exactive Orbitrap Mass Spectrometer (Thermo Fisher Scientific, Dreieich, Germany) equipped with a DESI source (Prosolia Inc., Zionsville, IN, USA) using an in-house built DESI sprayer in negative ionisation mode. The DESI was operated at a flow rate of 1 μL/min with 95:5 methanol/H_2_O. Nitrogen nebulising gas was operated at 4 bar. Images were acquired at a 20 μm spatial resolution and acquired with an MS scan range of 80–900 *m*/*z*, achieving 70,000 FWHM resolution. 

ThermoRAW files were converted to mzML format using MSConvertGUI with MS peak picking. Each row mzML file was converted to an image, imzML file, using the imzML Convertor programme [10,35]. Images were processed using SCiLS Labs Software version 2024b Pro (Bruker Daltonics, Bremen, Germany) following the selection of *m*/*z* values based on their statistical significance (*p* < 0.05) versus the magnitude of fold change from control to doxorubicin-treated MCTSs. Mean intensities of ions of interest were normalised to the total ion count (TIC) and graphed using GraphPad Prism software version 8.1.1 (GraphPad Prism, MA, USA). Unpaired parametric *t* tests were carried out on control and doxorubicin-treated comparisons, with Welch’s correction applied, whereby equal standard deviations between populations were not assumed. *p* values were annotated as follows and calculated at a 95% confidence interval: *p* = 0.1234 (ns), 0.0332 (*), 0.0021 (**), 0.0002 (***), and 0.0001 (****). Putative assignments were made with corresponding molecular formulae and mass errors detailed in Table S1.

### 2.6. Matrix-Assisted Laser Desorption Ionisation–Immunohistochemistry (MALDI-IHC)

MALDI-IHC was performed following the staining of 3D cell culture microtissue sections with a photocleavable mass tag (PC-MT) (AmberGen Inc., Boston, MA, USA) probe for Ki67. The previously reported manufacturer’s protocol [13] was followed, whereby microtissues were fixed, followed by washing steps to remove unfixed endogenous organic compounds. Rehydration was carried out, followed by antigen retrieval at alkaline pH. Blocking was carried out prior to the incubation of tissue sections with the photocleavable mass tag working probe solution, which consisted of 2.5 μg/mL Ki67 PC-MT. Tissues were vacuum-desiccated before the mass tags were photocleaved by UV illumination in the AmberGen Light Box. α-Cyano-4-hydroxycinnamic acid (CHCA) matrix, prepared in 70:30:0.1 ACN/water/TFA at a concentration of 10 mg/mL, was applied using the automated TM Sprayer (HTX Technologies, Chapel Hill, NC, USA) applied with 8 passes and a 2 mm track spacing at 75 °C, 414 millibar of gas pressure, an 80 μL/min pump flow rate, and 1100 mm/min nozzle velocity. Matrix recrystallization was carried out prior to analysis. MALDI-IHC was performed using a Rapiflex time-of-flight (TOF) mass spectrometer (Bruker Daltonics, Bremen, Germany). The laser was operated at a repetition rate of 10 kHZ and in positive ionisation mode. Imaging was performed at a spatial resolution of 10 μm with 300 laser shots per pixel and 50% laser power. The instrument was operated in reflector mode and scanned the mass range of 800 *m*/*z* to 1600 *m*/*z*. Images were processed using SCiLS Labs Software (Bruker Daltonics, Bremen, Germany) and were normalised to the TIC. Mean intensities across treatment groups were graphed using GraphPad Prism software version 8.1.1 (GraphPad Prism, Boston, MA, USA). Standalone pairwise comparisons between treatment groups were made by one-way ANOVA of mean intensities. *p* values were annotated as follows and calculated at a 95% confidence interval: *p* = 0.1234 (ns), 0.0332 (*), 0.0021 (**), 0.0002 (***), and 0.0001 (****).

### 2.7. Imaging Mass Cytometry by Time-of-Flight (IMC ToF)

Metabolite and protein distributions were correlated with complementary single-cell protein localisation using IMC ToF for the detection of proteins stained with metal-tagged antibodies at a subcellular 1 μm spatial resolution. IMC staining of the sectioned microtissues was carried out as previously described [8]. Briefly, the tissues were fixed with PFA, permeabilization was carried out with a Casein + 0.1% Triton X-100 solution, and tissues were blocked in Casein, before an incubation with the antibody cocktail in a humidified chamber at 4⁰C overnight. Conjugated antibodies were diluted in the Fluidigm antibody diluent (Standard BioTools, South San Francisco, CA, USA) as detailed in Table 1. Metal tag conjugations and the corresponding antibody targets used are also detailed in Table 1.

Following the antibody incubation, the Fluidigm DNA intercalator was applied and tissues were washed and air dried before IMC analyses were performed. Images were acquired by the Hyperion Imaging System (Standard BioTools, South San Francisco, CA, USA), which employs laser ablation coupled to the Helios™ inductively coupled plasma torch, and separation and detection were performed with the ToF mass analyser. Imaging data were extracted using MCD Viewer software v1.0.560.6 (Standard BioTools, South San Francisco, CA, USA). The quantitative analysis of IMC images was carried out using the HALO AI imaging platform (Indica Labs, Albuquerque, NM, USA). Cellular analysis was carried out to determine cell counts across models and was used to calculate the percentage of positive cells for each protein marker. Percentages across treatment groups were graphed using GraphPad Prism software version 8.1.1 (GraphPad Prism, Boston, MA, USA). Standalone pairwise comparisons between treatment groups were made by one-way ANOVA of mean percentages. *p* values were annotated as follows and calculated at a 95% confidence interval: *p* = 0.1234 (ns), 0.0332 (*), 0.0021 (**), 0.0002 (***), and 0.0001 (****). False discovery rate (FDR) analysis was performed on all *p* values using the original FDR method of Benjamini and Hochberg. A stringent minimum FDR threshold (q-value ≤ 0.05) at which comparisons were considered significant discoveries was applied.

### 2.8. Segmentation Analysis

DESI-MSI data were processed using SCiLS Labs Software (Bruker Daltonics, Bremen, Germany) where images were normalised to the TIC before the segmentation analysis was performed by applying a bisecting K-means algorithm. Partitional and hierarchical clustering were applied to identify distinct regions within the MCTSs. A segmentation analysis of DESI-MSI data was also performed using the Waters Software Labs MSI Segmentation Microapp version 2.1.0 (Waters Corporation, Wilmslow, UK). The computational dimension reduction technique Uniform Manifold Approximation and Projection (UMAP) was performed to identify distinct clustering patterns representative of distinct regions within the tumour models.

### 2.9. Discriminatory Analysis

Treatment-specific region of interest (ROI) feature lists were exported to MetaboAnalyst v5.0 [36]. An unsupervised multivariate analysis was performed to generate a principal component analysis (PCA) 2D plot with mean centring scaling applied. A component analysis was also carried out in SCiLS Labs Software (Bruker Daltonics, Bremen, Germany), whereby the values of each feature were mean centred and divided by the standard deviation of the feature to apply unit variance scaling to a principal component analysis 3D plot.

## 3. Results and Discussion

### 3.1. Characterisation of SAOS-2 MCTSs by DESI–MSI Revealed Tumour-Region-Specific Metabolism

**Figure 1:**
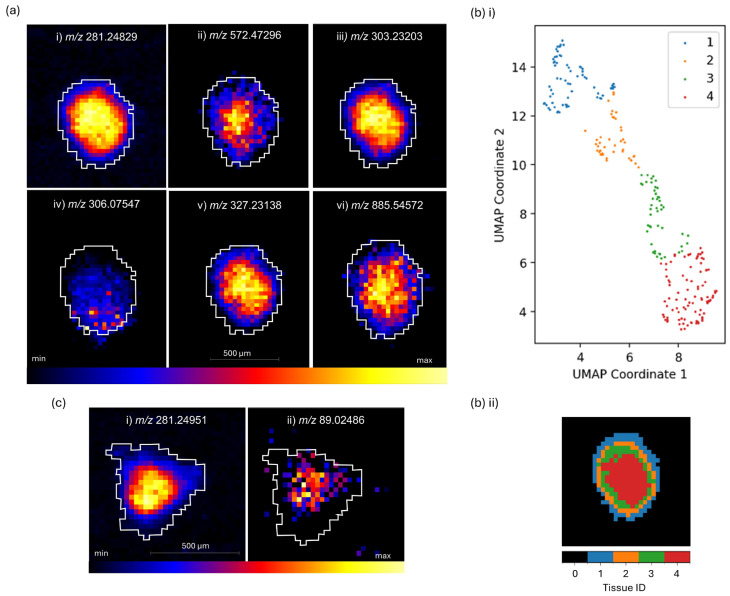
DESI-MSI of an SAOS-2 osteosarcoma Multicellular Tumour Spheroid acquired on the MRT MS in negative ionisation mode at 30 μm spatial resolution. (**a**) Ion density maps of (i) *m*/*z* 281.24829 (FA 18:1, e.g., oleic acid (1.10223 ppm)), (**ii**) *m*/*z* 572.47296 (C16 ceramide (d34:1) (14.91751 ppm)) (**iii**) *m*/*z* 303.23203 (FA 20:4 (3.19886 ppm)), (**iv**) *m*/*z* 306.07547 (glutathione (GSH) (3.36517 ppm)), (**v**) *m*/*z* 327.23138 (FA 22:6 (4.95060 ppm)), and (**vi**) *m*/*z* 885.54572 (PI 38:4 (4.72023 ppm)). (**b**) MSI tissue segmentation identified 4 distinct regions of the MCTS: (**i**) characterization by UMAP demonstrated 4 distinct clustering patterns that are representative of (**ii**) an outer proliferative region, two annular quiescent regions, and a hypoxic core. (**c**) Ion density maps of (**i**) *m*/*z* 281.24951 (FA 18:1 (3.23557 ppm)) (**ii**) *m*/*z* 89.02486 (lactate (5.16712 ppm)).

Ion density maps of MS images (Figure 1) revealed characteristic distributions to that of an in vivo tumour, whereby distinct localisation of lipids and metabolites were observed following DESI-MSI of SAOS-2 osteosarcoma MCTS models, acquired on the DESI MRT MS at a 30 μm spatial resolution in negative ionisation mode. A graduated increasing distribution towards the core of the spheroid was observed at *m*/*z* 281.24829 for FA 18:1, e.g., oleic acid (1.10 ppm), *m*/*z* 303.23203 for FA 20:4, e.g., arachidonic acid (AA), and *m*/*z* 327.23138 for FA 22:6, e.g., docosahexaenoic acid (DHA) (Figure 1a(i,iii,v)) and presented enhanced activity of fatty acid biosynthesis within the core of the tumour model. This indicated a metabolic change that could be attributed to increased cancer cell plasticity within the core under metabolically stressed conditions [37]. An increased fatty acid abundance is known to favour tumorigenesis and contribute to pro-oncogenic adaptations that support cancer cell survival. The upregulation of extracellular fatty acid uptake in particular has been linked to a hypoxic environment [38] and can be supported by the increased presence of the fatty acids identified here and localised within the hypoxic core of the MCTS (Figure 1a). Extracellular fatty acid uptake has been of particular interest in recent years with key candidates in anti-cancer agent drug development to include those that target fatty acid translocase, CD36 (cluster of differentiation 36), a key transporter of exogenous fatty acids into cancer cells [39]. Pre-clinical studies of CD36 inhibitors have shown promising signs of reduced cancer progression and metastasis [39], but have yet to progress a viable candidate to late-stage clinical trials [40].

The increased localisation of *m*/*z* 303.23203 (FA 20:4, e.g., AA) in the tumour model core (Figure 1a(iii)) is of interest given that AA, a polyunsaturated fatty acid (PUFA), and its metabolism are focal points of many drug development targets due to the implications for AA and its metabolites in pathways such as mTOR [41], MAPK [42,43] and PI3K [44,45]. A recent study found an upregulation of AA to be a target of OS with promising potential to chemosensitise OS cells given that far upstream element-binding protein 1 (FUBP1) was shown to be upregulated in OS cells and associated with a poorer clinical prognosis [46]. FUBP1 upregulates the AA metabolic pathway, and therefore AA and its metabolites are potential targets for increasing chemosensitivity and overcoming chemoresistance in OS. An increased chemoresistance of cancers with increased AA levels further supports the localisation within the core of the MCTSs that we see here and presents further insights into the contributing factors of chemoresistance mechanisms within the core of solid tumours. Targeting the AA metabolic pathways and associated prostaglandin cyclooxygenase (COX) metabolites has great chemotherapeutic potential [47], with an example of one of those metabolites, prostaglandin E2 (PGE2), being known to have high tumorigenic and metastatic potential and the ability to inhibit apoptosis [48,49], and it is therefore believed to largely contribute to chemoresistance.

A similar distribution was observed for *m*/*z* 885.54572 (PI38:4) (Figure 1a(vi)), which showed a graduated increasing distribution towards the core of the tumour model. The observed distribution links to the previously discussed association of AA and its implications for the PI3K pathway. The PI3K pathway and its activation are known to promote tumour growth and chemoresistance, and so it has been a widely researched drug target to develop inhibitors [50]. Phosphatidylinositols are parent compounds to the detected glycerophosphoinositol species, PI38:4, and therefore its increased localisation in the core of the MCTS can be related to an increase in PI3K pathway activation and chemoresistance. Other less abundant PI species (PI 34:1, PI 36:4, and PI 38:5), were also detected with distributions similar to PI38:4 (data not reported here). The significance of PI species in OS tumours is further supported by the evidenced sensitization of OS cells to radiation following exposure of a PI3K inhibitor [51]. Importantly, this sensitization effect was only observed in quiescent MG-63 cells and not proliferative MG-63 cells and therefore supports the localisation observed here, whereby the PI species was more highly localised in the quiescent region of the MCTS in comparison to the outer proliferative edge (Figure 1a(vi)).

An interesting localisation of *m*/*z* 572.47296 (C16 ceramide (d34:1)) was observed and indicates the likely presence of necrosis within a proportion of cells within the MCTSs, with greater abundance in the core of the model (Figure 1a(ii)). An additional ceramide species, Cer(d42:2), was also detected and presented a core localisation similar to that of the Cer(d34:1) presented in Figure 1a. However, Cer(d42:2) was of lower abundance and is not reported here. Cer(d34:1) has been identified as a biomarker for differentiating between viable and necrotic tumour regions within clinical breast cancer samples, whereby the ceramide species Cer(d34:1) was exclusively present in necrotic regions and undetectable in the viable tumour regions [52]. Necrosis is known to be associated with a poor prognosis for many tumour types due to its association with increased metastatic potential, angiogenesis and proliferative capabilities [53,54,55], and so our evidence here of the necrotic marker, *m*/*z* 572.47296, with the OS MCTS model presents a useful tool for investigating the tumour models’ responses to treatment and the role of necrosis in OS progression (Figure 1a(ii)). Necrosis in 2D cultures of OS cell lines has been associated with an upregulation of high-mobility group box 1 protein (HMGB1) [56], which is reported as a driver of chemoresistance within OS [57]. Despite this, an evaluation of necrosis within treatment-naïve and acutely treated OS is poorly experimented, likely since most assessments of OS necrosis are performed histologically via post neoadjuvant chemotherapy and surgical resection in order to evaluate the efficacy of the neoadjuvant chemotherapy in the clinic. The tumours are therefore more commonly at a stage of high necrosis, where a greater necrotic percentage is associated with more successful treatment outcomes and greater disease-free survival rates [58]. As a result, research of necrosis and its associated hypoxic environment within treatment-naïve OS and the impact on tumour progression is lacking. This work and the findings presented on the 3D MCTS models here, and previous studies that also detected Cer(d34:1) within the core of another type of OS 3D tumour model, the aggregoid model [9], begin to bridge the gap between simple 2D in vitro experiments that lack spatially resolved information in the context of tumour progression, and post neoadjuvant chemotherapy followed by surgical resection and late necrotic end point readouts.

A more homogenous distribution throughout the whole MCTS was observed for *m*/*z* 306.07547 (glutathione (GSH)) in Figure 1a(iv). Colocalisation with the fatty acid, phospholipid and ceramide species was not observed. GSH is known to counteract reactive oxygen species (ROS)-induced DNA oxidation and DNA damage [59]. It is upregulated to protect against increased ROS levels and subsequent oxidative stress that induce apoptosis [60]. The observed distribution demonstrates a relatively homogenous distribution of GSH throughout the MCTS model (Figure 1a(iv)) in comparison to a distinctive outer edge localisation, which was observed in the larger aggregoid OS tumour model [9]. The outer localisation of GSH could be due to the availability of nutrients, since GSH synthesis is reliant on the availability of sufficient amino acids, ATP and GCL and GC enzymes [61]. Additionally, by comparison, the smaller MCTS may reach a threshold of sufficient basic resources to synthesize GSH than the nutrient gradient allows in the aggregoid model.

Tissue segmentation of the OS MCTS demonstrated four distinct clustering patterns representative of tumour regions with distinct metabolic activity. Tissue classification identified clusters corresponding to four distinct tumour regions representative of tumour regions with different metabolic activities: an outer proliferative region, two annular quiescent regions, and a hypoxic core (Figure 1b(ii)). The computational dimension reduction technique, UMAP (Figure 1b(i)), following DESI–MSI, allowed the characterisation of these regions. Regional characterisation showed that the characteristics of the model were similar to that of an in vivo tumour microenvironment and demonstrated the clinical relevance of the MCTS tumour model.

Further DESI–MSI of the OS MCTSs revealed tumour-region-specific cancer metabolism in Figure 1c. Comparable distributions were observed between *m*/*z* 281.24951 (FA 18:1, e.g., oleic acid) (Figure 1c), and the glycolysis metabolite at *m*/*z* 89.02486 (lactate) which localised exclusively within the core of the MCTS (Figure 1c). The localisation of lactate here supports the well-established understanding that enhanced glycolysis is favoured over the TCA cycle in hypoxic tumour conditions [62]. Consequently, this finding has enabled the demonstration of this tumour survival mechanism, which is a characteristic of in vivo tumours. Pinpointing this mechanism in 3D cell culture tumour models by DESI-MSI underscores the increasing value of this approach as a tool to investigate drug resistance.

### 3.2. Chemotherapeutic-Induced Response of OS MCTSs by DESI-MSI

**Figure 2:**
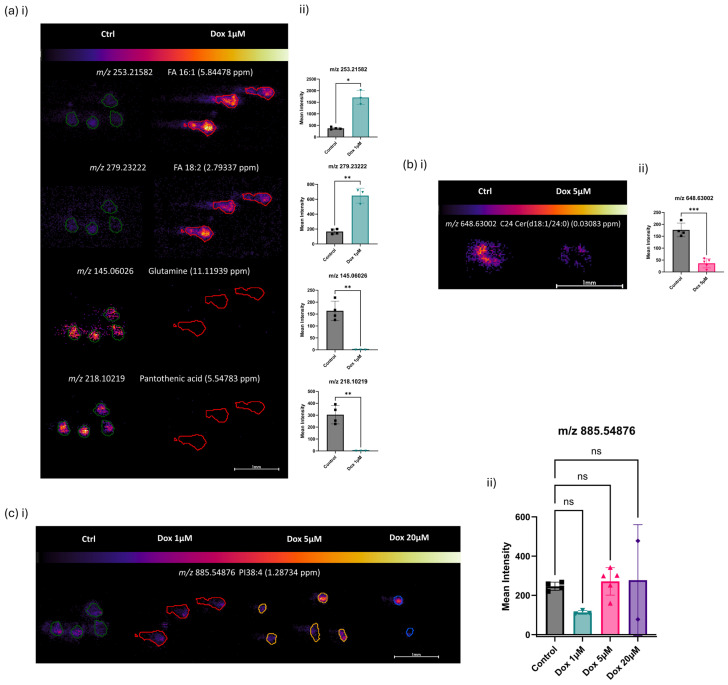
DESI-MSI of SAOS-2 Multicellular Tumour Spheroids following 48 h of exposure to doxorubicin doses. Images were acquired on the Orbitrap Q-Exactive mass spectrometer in negative ionisation mode at 20 μm spatial resolution. Outlines shown for control MCTSs (green), 1 μM Dox-treated MCTSs (red), 5 μM Dox-treated MCTSs (yellow) and 20 μM Dox-treated MCTSs (blue) are representative of tissue regions identified by partitional and hierarchical clustering following the application of a k-means bisecting algorithm. (**a**) (**i**) Ion images display increased abundances of *m*/*z* 253.21582 (FA 16:1, e.g., palmitoleic acid (5.84 ppm)) and *m*/*z* 279.23222 (FA 18:2, e.g., linoleic acid (2.79 ppm)) in 1 μM Dox-treated models. Additionally, decreased abundances of *m*/*z* 145.06026 (glutamine (11.12 ppm)) and *m*/*z* 218.10219 (pantothenic acid (5.55 ppm)) are shown in 1 μM Dox-treated models. (**ii**) Mean intensities of the corresponding ions of interest are presented, comparing the abundances in control and 1 μM Dox-treated MCTSs. (**b**) (**i**) Ion images display decreased abundances of *m*/*z* 648.63002 (C24 ceramide (d18:1/24:0) (0.03 ppm)) following 5 μM Dox exposure and displayed localisation to the outer proliferative edge of the MCTSs. (**ii**) Mean intensities of the corresponding ions of interest are presented. (**c**) (**i**) Ion images display a relatively consistent abundance of *m*/*z* 885.54876 (PI 38:4 (1.29 ppm)) across control, 1 μM, 5 μM and 20 μM Dox-treated MCTSs. (**ii**) Mean intensities of corresponding ions of interest are presented. *p* values obtained following unpaired parametric *t* tests are presented for comparisons between control and doxorubicin-treated MCTSs. *p* values = 0.1234 (ns), 0.0332 (*), 0.0021 (**), and 0.0002 (***) calculated at a 95% confidence interval.

Following the chemotherapeutic intervention, DESI-MSI followed by a receiver operating characteristic (ROC) curve analysis and principal component analysis (PCA)-guided ion density maps of produced images, significant molecular changes were identified. Figure 2 was acquired by negative ionisation DESI-MSI on the Orbitrap Q-Exactive mass spectrometer at 20 μm spatial resolution. Mean intensities of corresponding ions of interest are presented following the selection of *m*/*z* values based on their statistical significance (*p* < 0.05) versus the magnitude of fold change from control to 1 μM Dox treatment (Figure 2a(ii)). A significantly increased abundance within the tumour models upon dosage with 1 μM doxorubicin for 48 h (Figure 2a) was observed for *m*/*z* 253.21582 (FA 16:1, e.g., palmitoleic acid), and *m*/*z* 279.23222 (FA 18:2, e.g., linoleic acid (2.79 ppm)). This demonstrated an upregulation of fatty acid biosynthesis following the introduction of doxorubicin, demonstrating a previously undescribed mechanism of a doxorubicin-induced metabolic response within human osteosarcoma. As previously discussed for the characterisation of the MCTS models in Figure 1, an increased fatty acid presence has been widely reported to favour tumorigenesis and contribute to pro-oncogenic adaptations that support cancer cell survival [63,64]. Therefore, increased fatty acid biosynthesis has been hypothesised to be a potential chemoresistance mechanism. Yet, here, we report the first instance of the direct upregulation of FA species within OS following doxorubicin exposure. A study that investigated the hypothesis that the FA profile induces chemoresistance was able to demonstrate a decreased efficacy of doxorubicin in an obese mouse breast cancer model compared with lean mice [65]. These findings support the upregulation reported here (Figure 2a), with additional evidence of direct upregulation, independent of other variables, allowing the direct doxorubicin-induced response to be understood.

A contrasting relationship of the low-mass metabolites *m*/*z* 145.06026 (glutamine) and *m*/*z* 218.10219 (pantothenic acid) was observed following a 48 h cytotoxic treatment with 1 μM doxorubicin (Figure 2a). Glutamine is well understood to be crucial for supplying the metabolic reprogramming of cancer cells by feeding into the TCA cycle via glutaminolysis to support energy production under hypoxia [62]. A significant reduction in glutamine levels was observed within the OS MCTSs following 1 μM Dox treatments and therefore indicated a reduction in TCA cycle metabolic activity. A reduction in ATP production was further backed up by the cell viability assay presented later in the paperand supports the reduction in glutamine levels observed here (Figure 2a). Similarly, a significant downregulation of *m*/*z* 218.10219 (pantothenic acid) was observed following doxorubicin exposure. Pantothenic acid, also known as vitamin B5, was recently shown to colocalise with MYC-high tumour regions [66] and is therefore associated with proto-oncogenes that have high MCY expression levels and consequently more favourable metabolic regulation for malignant cancer progression [67]. A complementary reduction in both *m*/*z* 145.06026 (glutamine) and *m*/*z* 218.10219 (pantothenic acid) is supported by the understanding that MYC is known to upregulate glutaminolysis [68], and therefore the metabolically synergistic decrease following doxorubicin exposure is explained (Figure 2a).

Further discriminatory molecules of interest were identified by their attribution to a decreased abundance following doxorubicin exposure. OS tumour models subjected to 5 μM doxorubicin demonstrated a decreased abundance of *m*/*z* 648.63002 (C24 ceramide (d18:1/24:0)) (Figure 2b). A particularly interesting localisation of the ceramide was observed in the outer proliferative edge and extracellular region across both treatment groups. This distinct localisation was shown to become more predominantly localised only within the extracellular region of the MCTSs in the 5 μM doxorubicin-treated models (Figure 2b). Ceramides are understood to be key enriched components of extracellular vehicles (EVs) [69] and known to influence EV biogenesis [70]. Therefore, the localisation observed here gives a novel indication of the mechanisms of extracellular signalling and the roles they may play in osteosarcoma drug resistance, given their changes in localisation and decreased abundance following doxorubicin exposure (Figure 2b).

Considering the wider range of doxorubicin doses, control, 1 μM, 5 μM and 20 μM, at 48 h of exposure (Figure 2c), a consistent abundance of *m*/*z* 885.54876 (PI 38:4) was observed across the acutely treated MCTSs. Phosphatidylinositols are parent compounds to the detected glycerophosphoinositol species PI 38:4, and therefore its unchanged abundance with increasing doxorubicin dose could suggest unchanged PI3K pathway activation. The PI3K pathway and its activation are known to promote tumour growth and chemoresistance [50], whilst this distribution did not indicate any regulation of the pathway by doxorubicin. The insignificant change in the abundance of *m*/*z* 885.54876 (PI 38:4) across all treatment groups (Figure 2c(ii)) evidences a cellular component that remains predominantly unchanged across the tumour models (Figure 2c) and therefore supports the analytical robustness observed, given the upregulation and contrasting downregulation of metabolites observed in Figure 2a,b following the chemotherapeutic intervention. These DESI-MSI data aid in understanding the tumour model’s endogenous response to chemotherapy drug treatments and provide insights into the metabolic signatures of drug resistance.

### 3.3. A Discriminatory Analysis of OS MCTSs Revealed Doxorubicin-Dose-Dependent Separation of DESI-MSI Data

**Figure 3:**
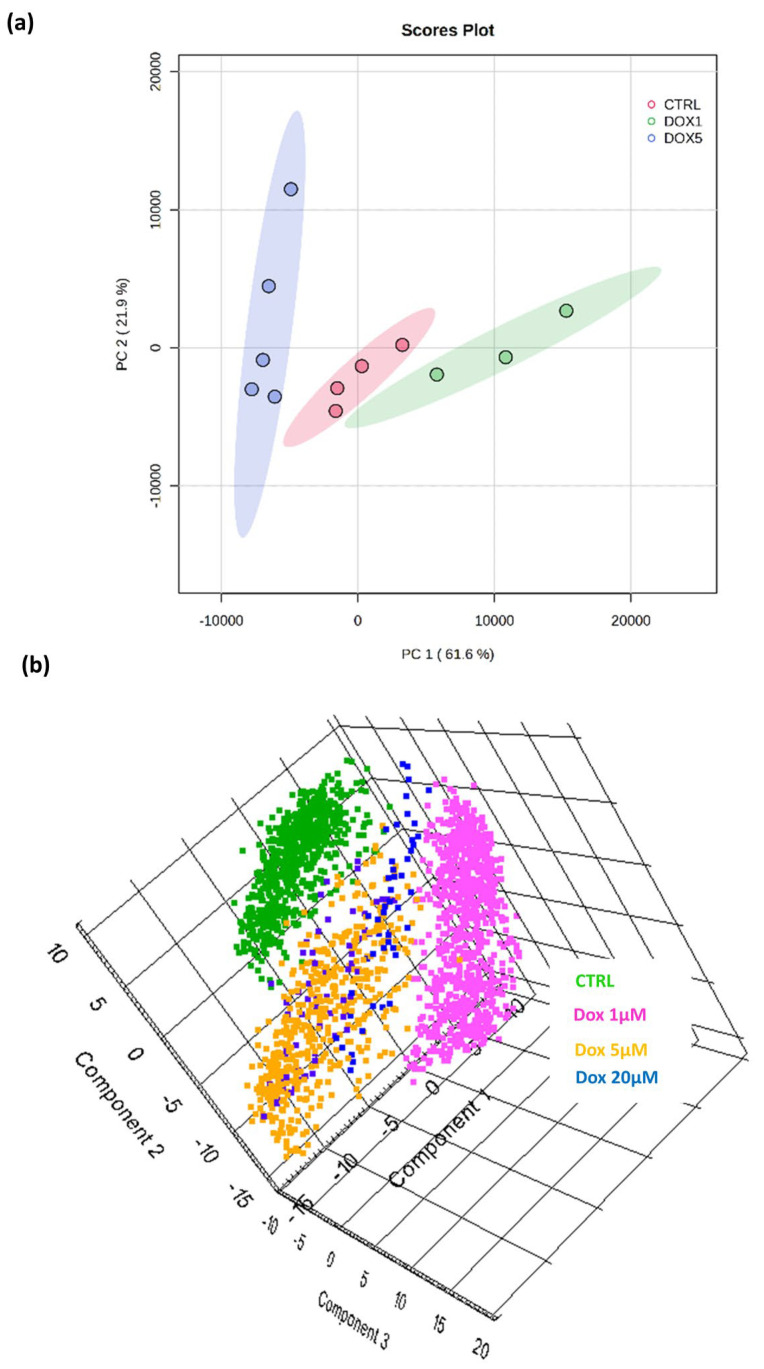
Principal component analysis (**a**) 2D and (**b**) 3D score plots presenting the significant separation of the molecular composition between (**a**) control SAOS-2 MCTSs and 1 μM, 5 μM (**b**) and 20 μM doxorubicin-treated MCTSs. Plots were generated from ROI feature lists exported to (**a**) MetaboAnalyst v5.0 with mean-centring scaling applied and (**b**) SCiLS Labs Software with unit variance scaling applied. PC1 (61.6%) and PC2 (21.9%).

By unsupervised multivariate analysis, principal component analysis (PCA) scores plots (Figure 3) demonstrated the dose-dependent discrimination of molecular composition between the control tumour models and those treated with increasing doxorubicin doses, which were shown in both the 2D and 3D PCA score plots.

The most variation in molecular composition was represented by a PC1 of 61.6% and the second most by a PC2 of 21.9% (Figure 3a); these data demonstrate that MSI of 3D tumour models offers the capability to further understand cancer progression, treatment responses, and drug resistance and is applicable to early drug discovery research by assessing the interplay of metabolic and cellular pathways in response to chemotherapeutic intervention.

### 3.4. Subcellular Protein Localisation Correlated with the Chemotherapeutic-Induced Metabolic Response of OS MCTSs by MALDI–IHC and IMC MSI

**Figure 4:**
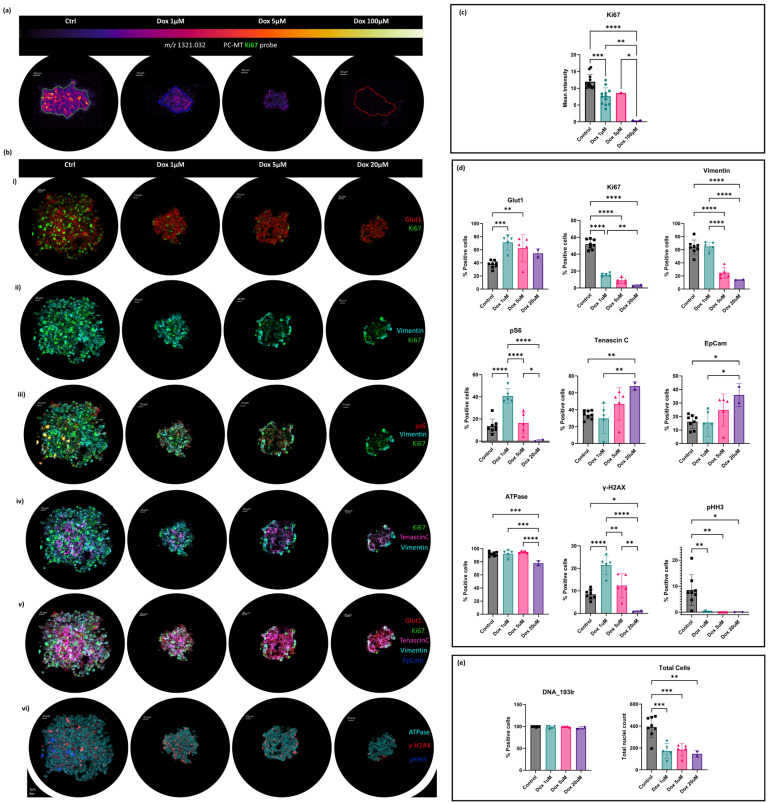
(**a**) MALDI-IHC of SAOS-2 Multicellular Tumour Spheroids following 48 h of exposure to the following doxorubicin doses: control, 1 μM, 5 μM, and 100 μM. Spheroids were stained with photocleavable mass tag antibodies for Ki67. The mass reporter *m*/*z* 1321.032 was detected in positive ionisation mode at 10 μm spatial resolution using the Rapiflex reflector TOF MS (Bruker Daltonics, Bremen, Germany). (**b**) Subcellular protein localisation IMC of SAOS-2 MCTSs following 48 h exposure to doxorubicin doses. Control and 1 μM, 5 μM, and 20 μM Dox-treated models were subjected to IMCyTOF MS following metal-tagged antibody staining and acquisition at 1 μm spatial resolution by the Hyperion Imaging System (Standard BioTools). Image overlays are presented for (**i**,**ii**) Glut1 and Ki67; (**iii**) ps6, Vimentin, and Ki67; (**iv**) Ki67, Tenascin-C, and Vimentin; (**v**) Glut1, Ki67, Tenascin-C, Vimentin, and EpCam; and (**vi**) ATPase, γ-H2AX, and pHH3. (**c**) Mean intensities of corresponding ions of interest are presented. *p* values obtained following one-way ANOVA are presented for comparisons between control and doxorubicin-treated MCTSs. (**d**) Percentages of positive cells for each protein marker are presented. *p* values obtained following one-way ANOVA are presented for comparisons between control and doxorubicin-treated MCTSs (**e**) Percentages of positive cells for DNA and total cell counts across all treatment groups are presented. *p* values obtained following one-way ANOVA are presented for comparisons between control and doxorubicin-treated MCTSs; *p* values = 0.1234 (ns), 0.0332 (*), 0.0021 (**), 0.0002 (***), and 0.0001 (****) calculated at a 95% confidence interval.

Here, the first application of MALDI-IHC to 3D cell culture models is reported (Figure 4a). These results support the recent introduction of MALDI-IHC into the field of mass spectrometry imaging [13], which has enabled high-multiplexing capabilities with absolute confirmatory protein identifications, whilst avoiding the typical roadblocks faced in a multiplexing, spatially resolved protein analysis. Whilst the multiplex analysis is not presented here, MALDI-IHC technology was employed to look at the expression of the proliferation marker Ki67 in the MCTSs by detecting the PC-MT probe at high spatial resolution (10 µm). MALDI-IHC of tumour models presented a significant reduction in the proliferation marker Ki67 with increasing doses of the chemotherapy drug, doxorubicin (Figure 4a,c). This enabled a confirmatory robust drug-induced response to be presented.

A complimentary approach was taken by the use of both MALDI-IHC and IMC, because although the use of IMC can present the localisation of Ki67 at near single-cell resolution, the data demonstrate the complimentary approach between the two methods and help to validate the findings of MALDI-IHC for future studies where a multi-omics approach can be applied for integrated spatial metabolomics and proteomics from the same MCTS section. An agreed reduction in cellular proliferation was also observed with a reduction in the level of the Ki67 marker with increasing dose following complimentary IMC (Figure 4b(i)). This allowed subcellular protein localisation utilizing metal-tagged antibodies and the acquisition of images on the Hyperion imaging system (Figure 4b(i–vi)), which were quantified by the percentage of positive cells for each protein marker across tumour model treatment groups (Figure 4d). Quantification of the Ki67 IMC marker demonstrated a mean of 52% proliferating KI67-positive cells in the control MCTSs compared to 16% in the 1 μM Dox-treated MCTSs. The 1 μM Dox-treated MCTSs therefore exhibited a 31% proliferative capability compared to that of the control MCTSs (Figure 4d). Ki67 protein expression occurs in all stages of the cell cycle but is known to vary throughout, with its maximum expression found in G2 phase or during mitosis [71]. This indicates that doxorubicin arrested further cell cycling at G2 phase and/or M phase, with Ki67-low cells remaining in MCTS doxorubicin treatment groups likely to be in G0, G1, or G1-S phases (Figure 4b(i)).

Glucose Transporter-1, GLUT1, an intrinsic hypoxia marker [72], was localised in the core of the control tumour model (Figure 4b(i)). This finding coincides with those presented following DESI-MSI of OS MCTSs in Figure 1c, whereby the tumour survival mechanism of increase glycolysis favoured over the TCA cycle was demonstrated with increased lactate abundance within the hypoxic core. Here (Figure 4b(i)), IMC demonstrated increased GLUT1 levels and subsequently increased glycolysis under hypoxic conditions within the core of the tumour model. The prominence of GLUT1 also significantly increased following doxorubicin exposure and therefore demonstrated increased hypoxia within the tumour models following the chemotherapeutic intervention (Figure 4d).

Vimentin, a structural filament protein, was evenly distributed throughout the control tumour (Figure 4b(ii)), as expected in osteosarcomas, and this can be attributed to its mesenchymal origin [73]. However, it localised to the outer edges of the MCTSs with increasing doses of doxorubicin (Figure 4b(ii)). This localisation is of interest since it is recognised that Vimentin is associated with accelerated tumour growth [74] and, since in the case of SAOS-2 cells, the distribution of Vimentin represents mesenchymal cells. Vimentin is known to be upregulated as a protective mechanism when stress is induced in other diseases [75], but this function is not well understood within cancer progression. Interestingly, rather than upregulation, what has been observed in this study is the downregulation and an absence of Vimentin in the core, with only an outer edge presence of Vimentin remaining with increasing doses of doxorubicin (Figure 4b.ii). This suggests that following the introduction of doxorubicin, a significant loss of mesenchymal characteristics was found in all regions other than the outer edge as a result of the introduction of the chemotherapeutic agent.

Ribosomal protein S6 (pS6) was almost absent in the control MCTSs but showed increased expression in MCTSs treated with 1 μM and 5 μM Dox compared to the control (Figure 4b(iii)). pS6 is an active downstream marker of the mammalian target of rapamycin (mTOR) pathway, and the upregulation of pS6 indicates a change in processes regulated by the mTOR pathway, including cancer cell progression, nutrient supply, and metastasis [76]. Changes in the expression of pS6 across the cell cycle are poorly understood within cancer progression, but a study using flow cytometry experiments reported that pS6-low cells were present only in G1 phase of the cell cycle, whereas pS6-high cells were present in G1, S, and G2 phases [77]. Mutant BRAF is known to constitutively activate downstream kinases of the mitogen-activated protein kinase (MAPK) pathway, which triggers uncontrolled cell proliferation [78]. In the study, pS6-low cells responded to BRAF drug inhibition and were arrested at G1 phase, whereas pS6-high cells did not respond and were distributed across G1, S, and G2 phases, like that of the untreated cells [77]. This response is consistent with the response observed in this work by IMC (Figure 4b(iii)), with pS6-low cells being arrested at G1 phase, whilst pS6-high cells did not respond as highly to doxorubicin. The pS6 abundance therefore displayed significantly increased dominance in the 1 μM doxorubicin-treated MCTSs compared to the control MCTSs (Figure 4d). As a result, the presented data suggest that pS6 could discriminate doxorubicin non-responding/resistant cells and hence could therefore be a novel biomarker of doxorubicin drug resistance.

A glycoprotein of the extracellular matrix (ECM), Tenascin-C, was found to be consistently localised in the core of the MCTSs across Dox treatment groups (Figure 4b(iv)). An exclusive core localisation demonstrates a relationship of increased Tenascin-C levels under hypoxic conditions, indicating a potential rise in hypoxia-related drug resistance. Tenascin-C is known to promote survival and invasion by regulating the expression of proangiogenic factors such as vascular endothelial growth factor (VEGF) [79]. The regulation of angiogenesis could play a key part in a localised tumour-core-specific drug resistance mechanism, demonstrating the role of Tenascin-C in the promotion of survival and expression of proangiogenic factors. The significant increase in the percentage of Tenascin-C-positive cells from the control and 1 μM Dox groups to the Dox 20 μM models presents interesting implications for the role of Tenascin-C in response to doxorubicin (Figure 4d). These findings indicate that Tenascin-C could serve as a potential target for tumour-core-specific drug resistance and high-dose doxorubicin resistance.

Furthermore, similar to Tenascin-C, the localisation of epithelial cell adhesion marker, EpCam, in the core of the control and Dox 1 μM dose groups was observed, and a distinctive drug-induced dispersion response with increasing doxorubicin dose was shown (Figure 4b(v)). The core localisation of EpCam could reveal the role the glycoprotein plays in solid tumour chemoresistance, as demonstrated not only by its core localisation but also its significant increase in expression within the higher dosed tumour models when comparing the percentage of EpCam-positive cells from control and 1 μM Dox models to the significant increase in 20 μM Dox models (Figure 4d).

ATPase showed unchanged expression within the MCTSs following exposure to 1 μM and 5 μM Dox (Figure 4b(vi)) and only showed a decrease in the percentage of ATPase-positive cells following exposure to a high doxorubicin dose of 20 μM (Figure 4d).

γ-H2AX displayed increased prominence in 1 μM Dox-dosed models, showing a 2.5-fold increase from the control and demonstrating the increased occurrence of double-strand breakage and the presence of DNA-damaged cells within the 1 μM Dox treatment group (Figure 4b(vi)). The 5 μM Dox model also displayed an increased presence of γ-H2AX and DNA damage compared to the control, but a smaller increase than the 1 μM Dox model, whilst the 20 μM Dox model displayed the smallest amount of DNA damage, expressing a low amount of γ-H2AX. The observed trend could be attributed to a differing mechanism of cell death between 1 μM Dox and the higher doses of 5 μM and 20 μM. At 1 μM, cytotoxicity is observed through Dox-induced DNA damage due to stalled replication forks and the induction of γ-H2AX. However, at the higher doxorubicin doses, cell death is likely not induced in the same way by DNA damage, rather a silencing of transcription factors and therefore increased expression of γ-H2AX is not observed (Figure 4b(vi),d). Supporting this, doxorubicin-induced cytotoxicity has been demonstrated by the increased expression of activating transcription factor 3 (ATF3) [80,81], suggesting that here we observed potential ATF3-induced cell death at higher doxorubicin doses. This suggests that cytotoxicity at the higher doses is induced by the upregulation of a phosphorylated extracellular signal-regulated kinase (pERK)-dependent pathway mediated by ATF3 and is interestingly independent of DNA damage and subsequent expression of γ-H2AX, therefore supporting the absence of increased γ-H2AX expression with increasing doses greater than 1 μM Dox (Figure 4b(vi),d).

The phospho-histone H3 (pHH3) mitotic marker was expressed in a low proportion of cells within the control MCTSs and was absent in all doxorubicin-treated models. This demonstrates reduced proliferation and a halt in mitotic activity following exposure to doxorubicin (Figure 4b(vi)). In comparison, a more gradual decrease in proliferative activity with increasing dose was observed with Ki67 (Figure 4b(i)). This difference is likely explained by pHH3 expression only occurring in mitosis and late G2 phases of the cell cycle in comparison to Ki67 expression across all stages of the cell cycle.

As expected, the presence of DNA was consistent across all MCTSs (Figure 4e). Nuclei counts demonstrated a significant two-fold decrease in the cell count in all doxorubicin-dosed MCTSs compared to the control MCTSs (Figure 4e). No significant change in cell count was observed with increasing doxorubicin concentrations from 1–20 μM. Interestingly, whilst the cell count was reduced two-fold, the mean area of the models was reduced four-fold following doxorubicin exposure, as demonstrated by IMC of the DNA marker in Supplementary Figure S1j(i). This interestingly suggests increased compactness of MCTS models following doxorubicin treatments.

All individual marker IMC data are presented in Figure S1a–j, and the false discovery rate (FDR) analysis is presented in Table S2.

### 3.5. Cell Viability of OS MCTSs Determined by the ATP Concentration and Correlated with the Doxorubicin-Induced Metabolic Response

**Figure 5:**
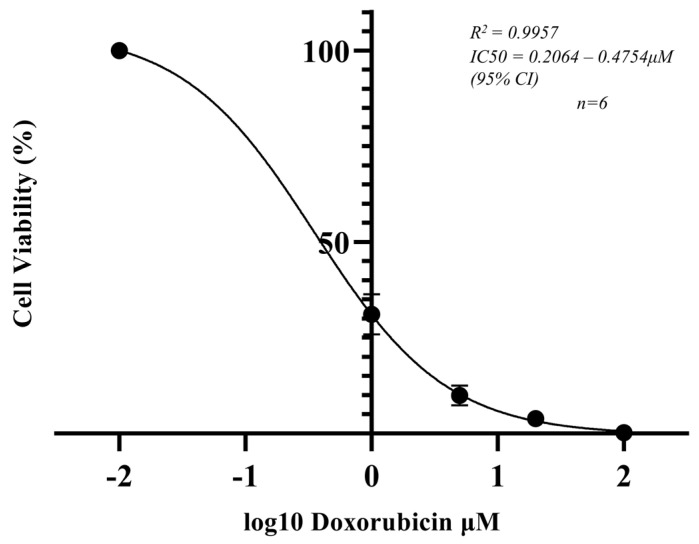
SAOS-2 MCTS dose–response curve following 48 h of exposure to 1 μM, 5 μM, 20 μM, or 100 μM doxorubicin. The response was determined by the CellTitre-Glo 3D Cell Viability Assay where Relative Luminescence Units (RLUs) were detected as a function of the ATP concentration. The inhibitor vs. normalised response was graphed in a non-linear regression fit (*n* = 6).

To further validate the metabolic response and change in protein localisation, the cell viability of MCTSs following doxorubicin exposure was measured. It was important to determine a cell viability dose response to provide context to the metabolic response and change in protein localisation. An SAOS-2 MCTS dose–response curve was produced following 48 h of exposure to 1 μM, 5 μM, 20 μM, or 100 μM doxorubicin. A response was determined by the CellTitre-Glo 3D Cell Viability Assay (Promega, UK), where Relative Luminescence Units (RLUs) were detected as a function of the ATP concentration [82] (Figure 5). A hypothesised relationship was observed, with a significant and reproducible response to doxorubicin across treatment groups. A significant reduction in ATP levels and a subsequent decrease in cell viability were observed. Cell viability decreased to 31% following 48 h of exposure to a 1 μM doxorubicin dose (Figure 5). The CellTitre-Glo by ATP read out presented superior percent coefficient of variation (%CV) values across repeats compared to that of commonly used cell viability assays to determine the dose response, including those observed by fluorescent microscopic dose response assays. The results present the ATP read out assay as an optimised dose response assay to precisely and reproducibly pair with the MSI and IMC drug-induced response of 3D cell culture tumour models of osteosarcoma. The viability data presented here (Figure 5) and those of further investigations involving varying chemotherapeutic agents demonstrate the capability to compliment mass spectrometry imaging, which allows for a wider understanding of the response with a largely untargeted approach to pinpoint changes in the molecular composition that indicate mechanisms of efficacy or resistance.

## 4. Conclusions

This study has employed a multi-modal mass spectrometry imaging approach, offering a label-free untargeted analysis of endogenous lipids and metabolites combined with single-cell-level spatial localisation and absolute protein identification to investigate the chemotherapy drug-induced response within acutely treated human osteosarcoma 3D cell culture tumour models. This approach was developed to satisfy the need for new approaches that can improve the efficiency of the drug discovery process. The development of in vitro tumour model dose response methodologies able to assess the interplay between metabolic and cellular pathways in response to chemotherapeutics will support steps towards the development of a more efficient drug development pipeline.

Mechanisms of action of doxorubicin drug resistance influenced by metabolomic changes in osteosarcoma remain largely unknown. This work presents previously unknown drug-induced molecular changes in response to doxorubicin treatment that inform doxorubicin mechanisms of action and suggest potential mechanisms of chemotherapeutic resistance. The significant increased abundance of the FA species presented in Figure 2a demonstrated an upregulation of fatty acid biosynthesis in response to doxorubicin, demonstrating a previously undescribed mechanism of the doxorubicin-induced metabolic response within human osteosarcoma by DESI-MSI. An increased fatty acid presence has been widely reported to favour tumorigenesis and contribute to pro-oncogenic adaptations that support cancer cell survival [63,64]. Yet, here, we report the first instance of a direct upregulation of FA species within OS following doxorubicin exposure.

Contrastingly, a significant downregulation of metabolites was observed within MCTSs following doxorubicin exposure, including glutamine, which demonstrated a reduction in glutaminolysis and consequently TCA cycle metabolic activity in response to doxorubicin. A reduction in ATP production was further evidenced by the dose–response curve presented in Figure 4 and complimented the reduction in glutamine levels observed by DESI-MSI (Figure 2a). Similarly, we present metabolite changes that indicate a downregulation of MYC-high proto-oncogenes that are known to favour metabolism and are advantageous to malignant cancer progression through the observed reduction in pantothenic acid levels following doxorubicin exposure (Figure 2a). The significance of the PUFA, AA, and its metabolism is considered surrounding its implications in pathways including mTOR [41], MAPK [42,43] and PI3K [44,45].

DESI-MSI has also provided a novel indication of mechanisms of extracellular signalling within 3D tumour models. Outer region localisation has given an initial indication of the roles that ceramide species-containing EVs may have in the osteosarcoma chemotherapy drug-induced response (Figure 2b). Additionally, the necrotic marker, *m*/*z* 572.47296 (Cer(d34:1)), localised in the core of the OS MCTSs and represents a useful tool for investigating drug-induced responses and for informing on the role of necrosis in OS progression (Figure 1a(ii)). Tumour survival mechanisms that are characteristic of those reported for in vivo tumours are also presented. Pinpointing the mechanism of enhanced glycolysis favoured over the TCA cycle under hypoxia in 3D cell culture tumour models by DESI-MSI (Figure 1c) underscores the increasing value of this approach as a tool to investigate drug resistance.

This study has also reported the first instance of MALDI-IHC MSI successfully executed on 3D cell culture models (Figure 4a,c). Whilst a multiplexed analysis was not presented here, MALDI-IHC allowed for absolute confirmatory protein identification following chemotherapy treatment of OS tumour models. Confirmatory absolute protein abundance changes were mapped at a high spatial resolution and demonstrated significant reductions in the cellular proliferation marker Ki67. Agreed conclusions of reduced proliferation following cytotoxic treatment were evidenced by complimentary IMC at subcellular spatial resolution (Figure 4b,d,e). Key findings include novel indications of a potential biomarker that selects for doxorubicin non-responding/resistant osteosarcoma cells presented by IMC analyses of pS6 within MCTSs acutely treated with 1 μM doxorubicin. Other indications for mechanisms of region-specific tumour survival were revealed by IMC, as well as cell-cycle-specific drug-induced responses, including indications of a significant loss of mesenchymal characteristics indicated by the localisation of Vimentin in all regions other than the outer edge following the introduction of the chemotherapeutic agent. IMC analysis also revealed indications for Tenascin-C as a potential target for tumour-core-specific drug resistance and high-dose doxorubicin resistance following its significantly increased prominence in 20 μM Dox models. Therefore, the results demonstrate the potential role of Tenascin-C in the promotion of survival and expression of proangiogenic factors within OS. An increased abundance of GLUT1 is present both within the core of the untreated tumour models compared to the outer edge of the MCTS but also in doxorubicin-treated models compared to the untreated models. Therefore, a demonstration of increased glycolysis within the hypoxic core and as a result of the chemotherapeutic intervention was shown. An interesting dose-dependent difference in the mechanism of cell death was presented for a lower dose of doxorubicin compared with the higher dose. This was shown by increased expression of γ-H2AX in models treated with the lower doses of doxorubicin, which was not observed in models treated with the higher doses. At higher doses, the data suggest that cell death is likely not induced in the same way by DNA damage, rather a silencing of transcription factors that is interestingly independent of DNA damage and subsequent expression of γ-H2AX.

Metabolic reprogramming of cancer tissues is widely studied and it is becoming better understood how cancer adapts to promote survival, proliferation and metastasis. However, direct metabolic reprogramming following chemotherapeutic intervention is less clear in the literature and therefore was addressed in this work. The application of sophisticated 3D cell culture models in the bioanalytical space is becoming pivotal for elucidating a wider aspect of cellular responses to therapeutic interventions and can now go beyond characterisation of the models. In this study, we have highlighted the significance of these advanced techniques in uncovering the dynamics of the drug response within the context of osteosarcoma when subjected to doxorubicin treatments. Without the need for animal testing, our approach provides improved clinical translatability compared to conventional in vitro studies, thereby elucidating potential strategies for pharmaceutical research.

The work allows us to address future perspectives whereby poor attrition rates of drug discovery are tackled. The approach of this study with a chemotherapeutic intervention and investigation of mass spectrometry imaging of 3D cell culture tumour models in combination with quantitative systems pharmacology (QSP) modelling is a perspective ideal future approach. Like that of R-based PBPK Simcyp simulations, as an automated high-throughput workflow [83]. A combination approach may be the way forward to create a more comprehensive early drug discovery approach.

## Figures and Tables

**Table 1:** Metal-conjugated antibody panel and the corresponding working concentrations prepared to stain microtissue sections.

Metal	Antibody Target	Clone	[Conjugated Ab]
143Nd	Vimentin	RV202	1:200
160Gd	GLUT1	EPR3915	1:100
166Er	EpCam	G8.8	1:100
167Er	Tenascin C	AZCYT0346/Poly	1:100
168Er	Ki67	B56	1:100
171Yb	ATPase	EP1845Y/AZCYT0253	1:100
175Lu	pS6	N7-548	1:250
173Yb	γH2AX	JBW301	1:50
176Lu	pHH3	HTA28	1:200

## Data Availability

The original data presented in the study are openly available in Sheffield Hallam University Research Data Archive SHURDA at https://shurda.shu.ac.uk/ accessed on 29 May 2024.

## Supplementary Materials

The following supporting information can be downloaded at https://www.mdpi.com/article/10.3390/metabo14060315/s1. Figure S1: Subcellular protein localisation IMC of SAOS-2 MCTSs following 48 h of exposure to doxorubicin doses. Control and 1 μM, 5 μM, and 20 μM Dox-treated models were subjected to IMCyTOF MS following metal-tagged antibody staining and acquisition at 1 μm spatial resolution by the Hyperion Imaging System. Individual protein marker images are presented for (a) (i) Glut1, (b) (i) Ki67, (c) (i) Vimentin, (d) (i) ps6, (e) (i) Tenascin-C, (f) (i) EpCam, (g) (i) ATPase, (h) (i) γ-H2AX, (i) (i) pHH3, and (j) (i) DNA. (a–j) (ii) Percentages of positive cells for each corresponding protein marker are presented. *p* values obtained following one-way ANOVA are presented for comparisons between control and doxorubicin-treated MCTSs. *p* values = 0.1234 (ns), 0.0332 (*), 0.0021 (**), 0.0002 (***), and 0.0001 (****) calculated at a 95% confidence interval. Table S1: DESI-MSI-identified mass list and corresponding mass errors. Table S2: False discovery rate (FDR) analysis of all IMC-produced *p* values when the abundance of detected protein markers was compared across MCTS treatment groups. The original FDR method of Benjamini and Hochberg was applied with a stringent minimum FDR threshold (q-value ≤ 0.05). Calculated q values and discovery classifications are presented.
