# Supplementary material for: Multimodal Mass Spectrometry Imaging of an Osteosarcoma Multicellular Tumour Spheroid Model to Investigate Drug-Induced Response
Source: Metabolites. 2024 May 29;14(6):315. doi: 10.3390/metabo14060315 (PMC11205347; doi:10.3390/metabo14060315)
Supplement: Supplementary file 1 [file metabolites-14-00315-s001.zip › metabolites-2995576-supplementary.pdf]

metabolites MDPI

Supplementary Materials

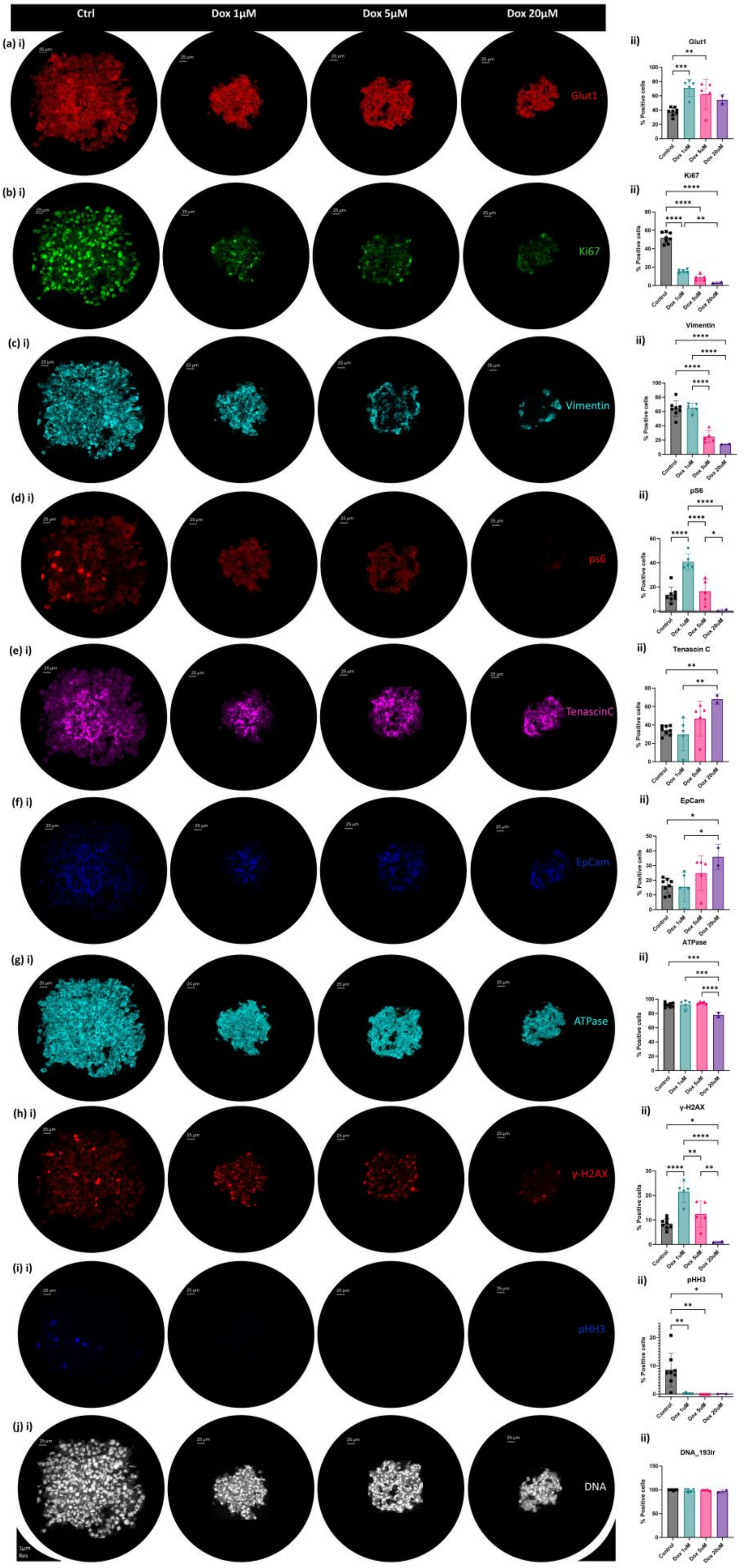

Supplementary Figure S1. Subcellular protein localisation IMC of SAOS-2 MCTSs following 48 hr of exposure to doxorubicin doses. Control and 1 μM, 5 μM, and 20 μM Dox-treated models were subjected to IMCyTOF MS following metal-tagged antibody staining and acquisition at 1 μm spatial resolution by the Hyperion Imaging System. Individual protein marker images are presented for **a) i)** Glut1, **b) i)** Ki67, **c) i)** Vimentin, **d) i)** ps6, **e) i)** Tenascin-C, **f) i)** EpCam, **g) i)** ATPase, **h) i)** γ-H2AX, **i) i)** pHH3, and **j) i)** DNA. **a-j) ii)** Percentages of positive cells for each corresponding protein marker are presented. P values obtained following one-way ANOVA are presented for comparisons between control and doxorubicin-treated MCTSs**.** P values = 0.1234 (ns), 0.0332 (*), 0.0021 (**), 0.0002 (***), and 0.0001 (****) calculated at a 95% confidence interval.

Supplementary Table S1. DESI-MSI-identified mass list and corresponding mass errors.

**Putative metabolite**	**Adduct**	**Formula**	***m/z* measured**	**Mass error (ppm)**	**Experiment**
FA 16:1	[M-H]-	C16H29O2-	253.21582	5.84	Figure 2. a.
FA 18:2	[M-H]-	C18H31O2-	279.23222	2.79	Figure 2. a.
Glutamine	[M-H]-	C5H9N2O3-	145.06026	11.12	Figure 2. a.
Pantothenic acid	[M-H]-	C9H16NO5-	218.10219	5.55	Figure 2. a.
C24 Ceramide (d18:1/24:0)	[M-H]-	C42H82NO3-	648.63002	-0.03	Figure 2. b.
PI38:4	[M-H]-	C47H82O13P-	885.54876	1.29	Figure 2. c.
FA 18:1	[M-H]-	C18H33O2-	281.24829	1.10	Figure 1. a.
C16 Ceramide (d34:1)	[M+Cl]-	C34H67ClNO3-	572.47296	14.92	Figure 1. a.
FA 20:4	[M-H]-	C20H31O2-	303.23203	3.20	Figure 1. a.
Glutathione (GSH)	[M-H]-	C10H16O6N3S-	306.07547	3.37	Figure 1. a.
FA 22:6	[M-H]-	C22H31O2-	327.23138	4.95	Figure 1. a.
PI38:4	[M-H]-	C47H82O13P-	885.54572	4.72	Figure 1. a.
FA 18:1	[M-H]-	C18H33O2-	281.24951	3.24	Figure 1. c.
Lactate	[M-H]-	C3H5O3-	89.02486	5.17	Figure 1. c.

Supplementary Table S2. False discovery rate (FDR) analysis of all IMC-produced P values when the abundance of detected protein markers was compared across MCTS treatment groups. The original FDR method of Ben-jamini and Hochberg was applied with a stringent minimum FDR threshold (q-value ≤ 0.05). Calculated q values and discovery classifications are presented.

**Variable Comparison**	**Discovery?**	**P value**	**q value**
Control vs. Dox5μM Vimentin	Yes	0.0001	0.0006
Control vs. Dox1μM Vimentin	No	0.8700	0.9642
Control vs. Dox20μM Vimentin	Yes	0.0001	0.0006
Dox5μM vs. Dox1μM Vimentin	Yes	0.0001	0.0006
Dox5μM vs. Dox20μM Vimentin	No	0.1743	0.2421
Dox1μM vs. Dox20μM Vimentin	Yes	0.0001	0.0006
Control vs. Dox5μM Glut1	Yes	0.0037	0.0100
Control vs. Dox1μM Glut1	Yes	0.0003	0.0012
Control vs. Dox20μM Glut1	No	0.1167	0.1821
Dox5μM vs. Dox1μM Glut1	No	0.2785	0.3561
Dox5μM vs. Dox20μM Glut1	No	0.4664	0.5868
Dox1μM vs. Dox20μM Glut1	No	0.1305	0.1958

Control vs. Dox5µM EpCam	No	0.0994	0.1685
Control vs. Dox1µM EpCam	No	0.8929	0.9673
Control vs. Dox20µM EpCam	Yes	0.0112	0.0282
Dox5µM vs. Dox1µM EpCam	No	0.1085	0.1801
Dox5µM vs. Dox20µM EpCam	No	0.1489	0.2191
Dox1µM vs. Dox20µM EpCam	Yes	0.0128	0.0312
Control vs. Dox5µM Tenascin C	No	0.1143	0.1821
Control vs. Dox1µM Tenascin C	No	0.5787	0.6839
Control vs. Dox20µM Tenascin C	Yes	0.0058	0.0151
Dox5µM vs. Dox1µM Tenascin C	No	0.0609	0.1159
Dox5µM vs. Dox20µM Tenascin C	No	0.0801	0.1453
Dox1µM vs. Dox20µM Tenascin C	Yes	0.0037	0.0100
Control vs. Dox5µM Ki67	Yes	0.0001	0.0006
Control vs. Dox1µM Ki67	Yes	0.0001	0.0006
Control vs. Dox20µM Ki67	Yes	0.0001	0.0006
Dox5µM vs. Dox1µM Ki67	No	0.0251	0.0529
Dox5µM vs. Dox20µM Ki67	No	0.1219	0.1864
Dox1µM vs. Dox20µM Ki67	Yes	0.0030	0.0087
Control vs. Dox5µM ATPase	No	0.1676	0.2377
Control vs. Dox1µM ATPase	No	0.8777	0.9642
Control vs. Dox20µM ATPase	Yes	0.0002	0.0010
Dox5µM vs. Dox1µM ATPase	No	0.2623	0.3413
Dox5µM vs. Dox20µM ATPase	Yes	0.0001	0.0006
Dox1µM vs. Dox20µM ATPase	Yes	0.0003	0.0012
Control vs. Dox5µM γ-H2AX	No	0.0826	0.1464
Control vs. Dox1µM γ-H2AX	Yes	0.0001	0.0006
Control vs. Dox20µM γ-H2AX	Yes	0.0222	0.0481
Dox5µM vs. Dox1µM γ-H2AX	Yes	0.0013	0.0048
Dox5µM vs. Dox20µM γ-H2AX	Yes	0.0021	0.0063
Dox1µM vs. Dox20µM γ-H2AX	Yes	0.0001	0.0006
Control vs. Dox5µM pS6	No	0.5024	0.6220
Control vs. Dox1µM pS6	Yes	0.0001	0.0006
Control vs. Dox20µM pS6	No	0.0392	0.0784
Dox5µM vs. Dox1µM pS6	Yes	0.0001	0.0006
Dox5µM vs. Dox20µM pS6	Yes	0.0197	0.0439
Dox1µM vs. Dox20µM pS6	Yes	0.0001	0.0006
Control vs. Dox5µM pHH3	Yes	0.0015	0.0051
Control vs. Dox1µM pHH3	Yes	0.0017	0.0053
Control vs. Dox20µM pHH3	Yes	0.0143	0.0328
Dox5µM vs. Dox1µM pHH3	No	0.9688	0.9999
Dox5µM vs. Dox20µM pHH3	No	0.9999	0.9999
Dox1µM vs. Dox20µM pHH3	No	0.9764	0.9999

Control vs. Dox5µM DNA 193	No	0.8534	0.9642
Control vs. Dox1µM DNA 193	No	0.0984	0.1685
Control vs. Dox20µM DNA 193	No	0.0770	0.1430
Dox5µM vs. Dox1µM DNA 193	No	0.1769	0.2421
Dox5µM vs. Dox20µM DNA 193	No	0.1167	0.1821
Dox1µM vs. Dox20µM DNA 193	No	0.5631	0.6805
Control vs. Dox5µM DNA 191	No	0.9921	0.9999
Control vs. Dox1µM DNA 191	No	0.1620	0.2340
Control vs. Dox20µM DNA 191	No	0.0362	0.0743
Dox5µM vs. Dox1µM DNA 191	No	0.2020	0.2717
Dox5µM vs. Dox20µM DNA 191	No	0.0457	0.0891
Dox1µM vs. Dox20µM DNA 191	No	0.2625	0.3413
Control vs. Dox5µM Total	Yes	0.0003	0.0012
Control vs. Dox1µM Total	Yes	0.0002	0.0010
Control vs. Dox20µM Total	Yes	0.0011	0.0043
Dox5µM vs. Dox1µM Total	No	0.8209	0.9416
Dox5µM vs. Dox20µM Total	No	0.5671	0.6805
Dox1µM vs. Dox20µM Total	No	0.6869	0.7997
